# Supplementary figures and images for: Land use management based on multi-scenario allocation and trade-offs of ecosystem services in Wafangdian County, Liaoning Province, China
Source: PeerJ. 2019 Sep 16;7:e7673. doi: 10.7717/peerj.7673 (PMC6752191; doi:10.7717/peerj.7673)

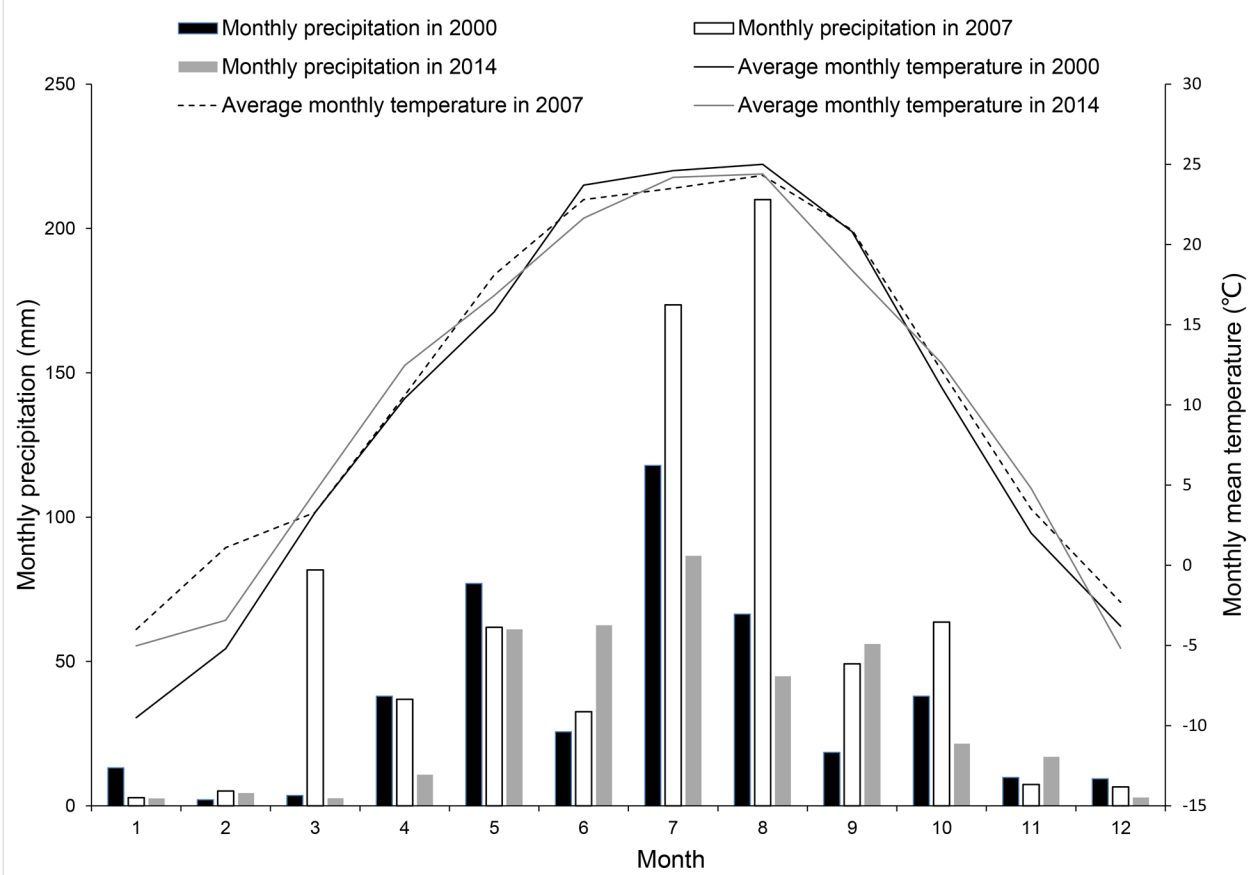

Supplement: Figure S1 [file peerj-07-7673-s001.pdf]

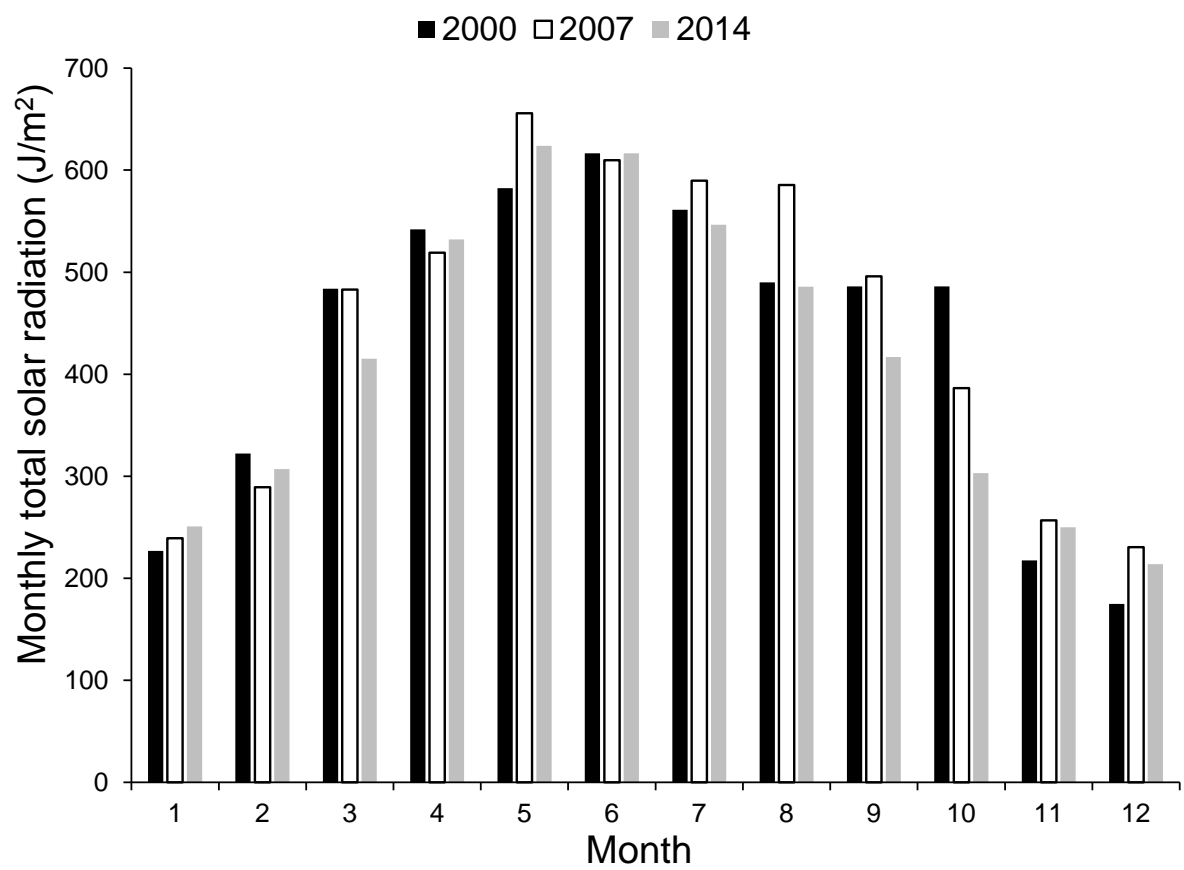

Supplement: Figure S2 [file peerj-07-7673-s002.pdf]

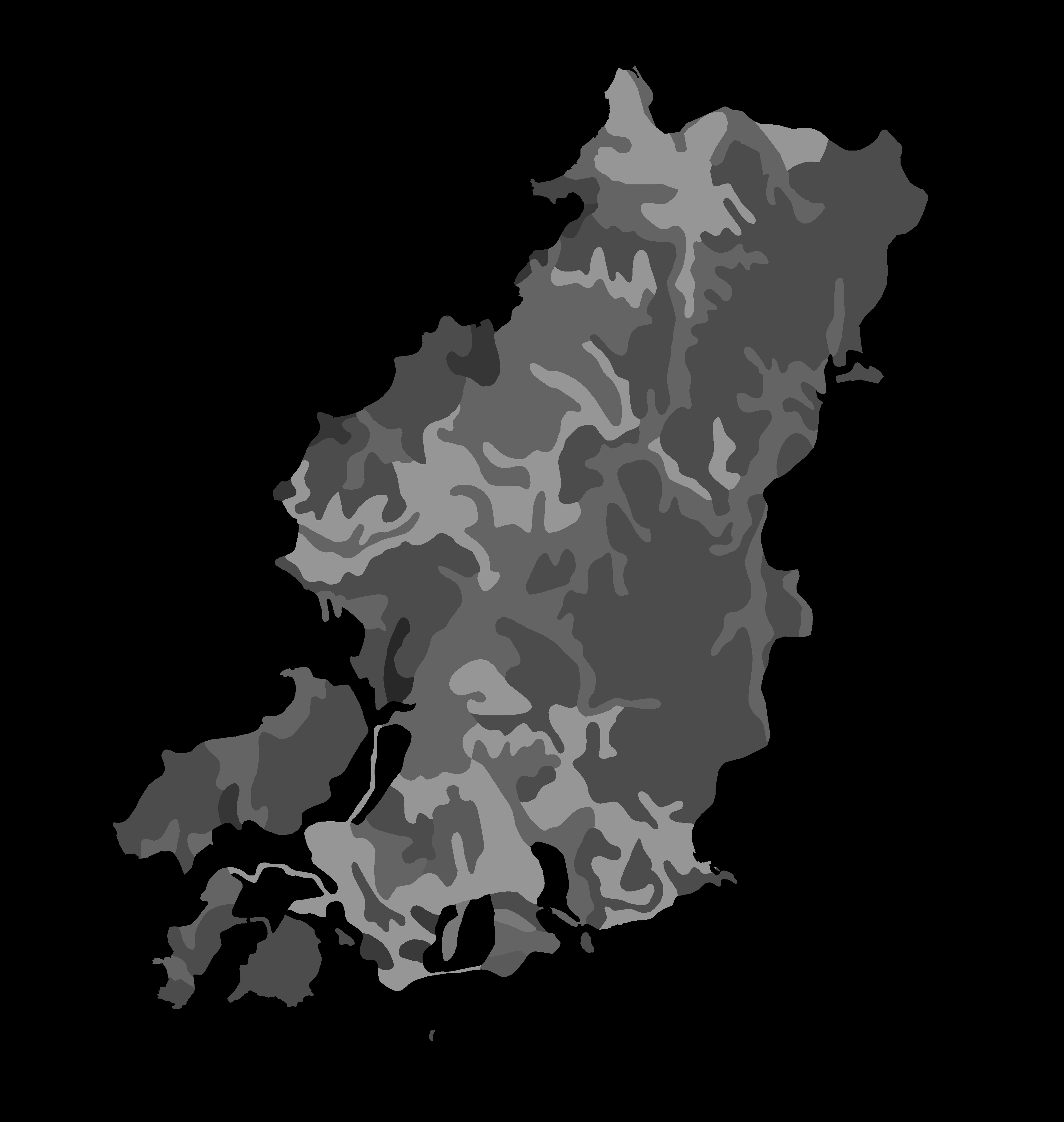

Supplement: Supplemental Information 2 [file peerj-07-7673-s005.zip › Key parameters/soildepth/soildepth.tif]
