# Supplementary material for: Land use management based on multi-scenario allocation and trade-offs of ecosystem services in Wafangdian County, Liaoning Province, China
Source: PeerJ. 2019 Sep 16;7:e7673. doi: 10.7717/peerj.7673 (PMC6752191; doi:10.7717/peerj.7673)
Supplement: Table S1 [file peerj-07-7673-s003.docx]

| Climate factors | 2000 | 2007 | 2014 |
| --- | --- | --- | --- |
| Monthly mean precipitation (mm) | 34.96 | 60.94 | 31.08 |
| Monthly mean temperature (℃) | 9.85 | 11.16 | 10.53 |
| Monthly mean total solar radiation (J/m^2^) | 413.44 | 445.08 | 432.47 |
